# Development of a clincally‐viable, single AAV vector for *App* C‐terminus CRISPR editing

**DOI:** 10.1002/alz.087033

**Published:** 2025-01-09

**Authors:** Brent D Aulston, Leonardo A Parra‐Rivas, Subhojit Roy, Donald Pizzo

**Affiliations:** ^1^ University of California San Diego, La Jolla, CA USA

## Abstract

**Background:**

Our lab has developed a CRISPR‐based, gene‐editing strategy that targets the extreme C‐terminus (C‐term) of APP (amyloid precursor protein) – a gene with a central and indisputable role in AD. We have reported previously that APP C‐terminus CRISPRs effectively attenuate APP β‐cleavage and Alzheimer’s pathology in vivo. Here, we present new data demonstrating the feasibility and efficacy of a clinically‐viable, “all‐in‐one” therapeutic vector that has all the components needed for APP C‐terminus editing (Cas enzyme / gRNAs / regulatory elements) packaged into a single AAV.

**Method:**

APP C‐terminus targeting gRNAs along with a SaCas9 expression cassette were cloned into AAV expression backbones and packaged into AAV‐PHP.eB capsids. Resulting AAVs were injected into APP^NL‐G‐F^ mice and APP C‐terminus editing along with amyloid beta (Aβ) plaque pathology were evaluated.

**Result:**

AAV treated brain tissue showed wide‐spread expression of APP CRISPRs and robust APP C‐terminus editing. Both amyloid plaques and insoluble Aβ were decreased in APP CRISPR treated tissue.

**Conclusion:**

These data demonstrate in vivo feasibility of our approach and advance our long‐term goal of creating a “one‐and‐done”, single injection CRISPR therapeutic for permanent treatment of AD in human patients.

